# The trend of neutrophil-to-lymphocyte ratio and platelet-to-lymphocyte ratio in spontaneous intracerebral hemorrhage and the predictive value of short-term postoperative prognosis in patients

**DOI:** 10.3389/fneur.2023.1189898

**Published:** 2023-05-25

**Authors:** Jian Zhang, Chunlong Liu, Yaofeng Hu, Aoran Yang, Yonghui Zhang, Yang Hong

**Affiliations:** ^1^Department of Neurosurgery, Shengjing Hospital of China Medical University, Shenyang, China; ^2^Department of Neurosurgery, The Seventh Clinical College of China Medical University, Fushun, China; ^3^Department of Hepatobiliary and Pancreatic Surgery, Fuyang People's Hospital, Anhui Medical University, Fuyang, China

**Keywords:** spontaneous intracerebral hemorrhage, neutrophil-to-lymphocyte ratio, platelet-to-lymphocyte ratio, surgery, prognosis, marker

## Abstract

**Background:**

Neutrophil-to-lymphocyte ratio (NLR) and platelet-to-lymphocyte ratio (PLR) play an important role in the inflammatory response in various diseases, but the role in the course of spontaneous intracerebral hemorrhage (ICH) is unclear.

**Methods:**

This study retrospectively collected baseline characteristics and laboratory findings, including NLR and PLR at different time points, from spontaneous ICH patients undergoing surgery between January 2016 and June 2021. Patients were scored using the modified Rankin Scale (mRS) to evaluate their functional status at 30 days post-operation. Patients with mRS score ≥3 were defined as poor functional status, and mRS score <3 was defined as good functional status. The NLR and PLR were calculated at admission, 48 h after surgery and 3–7 days after surgery, respectively, and their trends were observed by connecting the NLR and PLR at different time points. Multivariate logistic regression analysis was used to identify independent risk factors affecting the prognosis of ICH patients at 30 days after surgery.

**Results:**

A total of 101 patients were included in this study, and 59 patients had a poor outcome at 30 days after surgery. NLR and PLR gradually increased and then decreased, peaking at 48 h after surgery. Univariate analysis demonstrated that admission Glasgow Coma Scale (GCS) score, interval from onset to admission, hematoma location, NLR within 48 h after surgery and PLR within 48 h after surgery were associated with poor 30-day prognosis. In multivariate logistic regression analysis, NLR within 48 h after surgery (OR, 1.147; 95% CI, 1.005, 1.308; P, 0.042) was an independent risk factor for 30-day after surgery prognosis in spontaneous ICH patients.

**Conclusion:**

In the course of spontaneous intracerebral hemorrhage, NLR and PLR initially increased and subsequently decreased, reaching their peak values at 48 h after surgery. High NLR within 48 h after surgery was an independent risk factor for poor prognosis 30 days after surgery in spontaneous ICH patients.

## 1. Introduction

Cerebrovascular disease (CVD) is a significant cause of death and disability worldwide, including acute ischemic stroke (AIS) and intracerebral hemorrhage (ICH), mostly caused by acute arterial occlusion and rupture (1, 2). ICH occurs in 10–15% of all stroke types and places a huge economic burden on society and families (3). Spontaneous ICH is a non-traumatic hemorrhagic disease that develops in 10–30 per 100,000 people globally, affecting approximately 2 million people worldwide, and has a high mortality and disability rate (4). In selected patients surgery can be life saving. However, identifying the early prognosis in ICH patients after surgery is more difficult. At present, the indicators for prognostic evaluation in ICH patients after surgery focus on the general condition of the patient at admission, such as GCS score, pupil size, pupil reactivity, hematoma location, hematoma volume, and whether the hematoma extends into the ventricle. However, these prognostic indicators have different drawbacks (5).

Neutrophil-to-lymphocyte ratio (NLR) and platelet-to-lymphocyte ratio (PLR) are indicators of inflammatory response and coagulation status, which play an important role in the prognosis prediction of stroke. A larger cohort study revealed that pre-thrombolytic levels of NLR and PLR were significantly associated with early neurological deterioration following thrombolysis in patients with acute ischemic stroke (6). Furthermore, a recent retrospective study demonstrated the predictive value of elevated levels of NLR in both the development of stroke-associated pneumonia in ICH patients and the early identification of pneumonia severity (7). The study indicated that the neuroinflammatory response was associated with secondary brain injury after spontaneous ICH, thus the inflammatory markers NLR and PLR might be markers of prognosis in ICH patients. In the early stages of ICH, microglia activate and release chemokines to recruit peripheral inflammatory cells are the main cause of secondary brain injury (8). Over time, leukocyte infiltration increase in the injury site, releasing large amounts of inflammatory mediators, which may exacerbate secondary brain injury. In addition, activated platelets begin to promote the recruitment of leukocytes at the site of injury, further aggravating the injury (9). There is increasing evidence that NLR and PLR are more accurate compared to other markers. NLR and PLR can effectively assess early neurological deterioration and long-term survival of stroke patients (8, 10). Regarding the relationship between inflammatory indicators and ICH, current studies focused on a point of time or a period of time (11), ignoring the trend of inflammatory indicators over the course of the disease. Surgery is an important part of the therapy for spontaneous ICH, intervening in the disease process by effectively reducing intracranial pressure, relieving the hematoma-occupying effect and preventing the massive release of inflammatory mediators (12). Different surgical methods vary in the degree of hematoma removal, but equally differ in the degree of brain tissue damage. In Addition, it is controversial whether different surgical methods can improve the prognosis of spontaneous ICH patients (13, 14).

## 2. Methods

### 2.1. Study design

This study retrospectively collected clinical data from 101 spontaneous ICH patients undergoing craniotomy and minimally invasive puncture and drainage (MIPD) between January 2016 and June 2021. The Medical Ethics Review Committee of Liaoning Health Industry Group Fukuang General Hospital approved this study, which was conducted under the guidelines of the World Medical Association Helsinki Declaration in 1975. Informed written consent was obtained from all patients or their relatives.

Inclusion criteria: (1) age > 18 years old and first presentation; (2) spontaneous ICH confirmed by computed tomography (CT); (3) craniotomy or MIPD for hematoma drainage within 24 h of onset.

Exclusion criteria: (1) age < 18 years; (2) secondary ICH due to trauma, aneurysm, arteriovenous malformation, cavernous angioma, smoldering disease, tumor stroke, or coagulation disorders; (3) infectious diseases, cancer, chronic heart disease, liver or kidney disease, rheumatic immune system disease, hematologic diseases, and other diseases affecting peripheral blood cells; (4) patients without CT or angiography at 24 h after admission; (5) admission GCS score < 5, patients were paralyzed or in a vegetative state before the onset of the disease; (6) incomplete follow-up data.

### 2.2. Preoperative patient management

All patients included in this study were managed and treated according to the American Heart Association/American Stroke Management Guidelines (15). Upon admission, patients were monitored for vital signs, assessed for consciousness with GCS scores, and immediately underwent CT to assess the hemorrhage location, hematoma volume, and whether there was a combined ventricular hemorrhage and subarachnoid hemorrhage, while peripheral blood was collected. Two experienced neurosurgeons developed the surgical procedure based on the clinical situation of each ICH patient, including access location and bone window size.

### 2.3. Clinical parameter evaluation

We retrospectively collected demographic characteristics, general clinical characteristics, imaging findings, and laboratory findings of all ICH patients enrolled in this study, including age, sex, hypertension history, diabetes history, admission systolic blood pressure, admission diastolic blood pressure, admission GCS score, interval from onset to admission, hematoma location (supratentorial and infratentorial), hematoma volume (calculated using ABC/2 software) (16), ventricular hemorrhage, subarachnoid hemorrhage, and surgery methods (craniotomy and MIPD).

Laboratory test results including serum potassium ion, serum glucose, fibrinogen (FIB), prothrombin time (PT), activated partial thromboplastin time (APTT), thrombin time (TT), and D-dimer were collected from patients on admission. On admission, 48 h after surgery and 3–7 days after surgery white blood cell (WBC), absolute neutrophil (NE) count, absolute lymphocyte (L) count, and platelet (PLT) count were measured. NLR was defined as neutrophil count to lymphocyte count ratio and PLR was defined as platelet count to lymphocyte count ratio. NLR and PLR were assessed at admission, 48 h post-surgery, and 3–7 days post-surgery. NLR values were labeled as NLR1, NLR2, and NLR3, while PLR values were labeled as PLR1, PLR2, and PLR3, respectively. Both NLR and PLR values were considered continuous variables and were compared between the good and poor prognosis groups using Student’s t-test to identify potential differences across various time points. A statistically significant difference was established if *p* < 0.05.NLR and PLR were presented as mean ± standard deviation values. Line segments were used to connect NLR and PLR data points from different time points to analyze the trends of inflammatory indicators in all patients, craniotomy group patients, and MIPD group patients separately.

### 2.4. Surgery method

The craniotomy group routinely performed hematoma removal according to the preoperatively designed surgical approach and bone window size, aspirating the hematoma as cleanly as possible, and whether the bone flap was preserved or not depended on the intraoperative situation. The MIPD group punctured the hematoma according to the pre-designed direction, location and depth after installing and commissioning the stereotactic instrument, and slowly drained the blood using a 5 mL syringe.

### 2.5. Postoperative patient management

Standardized management of postoperative patients according to the American Heart Association/American Stroke Management Guidelines. In the MIPD group, we gave 5 mL of saline mixed with 20–30,000 units of urokinase every 4–6 h postoperatively in order to liquefy the hematoma. Regular cranial CT examinations were performed to assess the effect of hematoma removal, and the drainage tube was removed when the hematoma disappeared or the remaining hematoma volume was less than 10 mL and the patient’s vital signs were stable. Peripheral blood specimens were collected daily at 7:00 am after surgery. Patient prognosis was assessed using the mRS to acquire patients’ survival functional status and outcomes at 30 days after surgery with outpatient or telephone follow-up visits (17). Patients with mRs ≥3 defined as poor prognosis and mRs <3 as good prognosis at 30 days postoperatively.

### 2.6. Statistical analysis

Statistical analysis of the data was performed using SPSS 25.0 (IBM Corporation, Almonk, NY, USA). Graphs were plotted using GraphPad Prism 9.0 (GraphPad Software, San Diego, CA, USA). All continuous variables were expressed as mean ± standard deviation or median (interquartile range, IQR) and analyzed using Student’s *t*-test or Mann–Whitney test. All categorical variables were expressed as frequencies (percentages) and analyzed using chi-square tests. Variables included in the univariate analysis that were significant (*p* < 0.10) were entered into multivariate logistic regression and defined as independent risk factors for poor outcome. The receiver operating characteristic analysis (ROC) were used to assess different variables. *p* < 0.05 was considered to be statistically significant.

## 3. Results

### 3.1. ICH patients’ general information

In this study, a total of 101 spontaneous ICH patients (69 males and 32 females) with a median age of 59 years (IQR: 53.5–66 years) were included, 42 in the favorable prognosis group and 59 in the poor prognosis group (Table 1). The median time from patient onset to admission was 3 h (IQR: 2–5 h). 70% patients had hypertension history. The GCS score on admission was 9 (IQR: 8–12). Among all patients, 86 (84.2%) had hematoma locations supratentorial and 16 (15.8%) were infratentorial. Of all patients with supratentorial hematomas, 50 (49.5%) were located in the basal ganglia and 35 (34.7%) in the cerebral lobes. The hematoma volume was 40.0 mL (IQR: 28.3–60.6 mL). 24 patients (23.8%) had combined subarachnoid hemorrhage. All patients underwent surgical treatment, 77 (76.2%) underwent craniotomy and 24 (23.8%) underwent MIPD. Of all the ICH patients who underwent craniotomy, 8 had preserved bone flaps, 3 had basal ganglia hemorrhage, and 5 had lobar hemorrhage. Among the 3 patients with basal ganglia hemorrhage, 2 displayed poor functional status at 30 days post-operation, while 1 showed good functional status. Regarding the 5 patients with lobar hemorrhage, all of them had a good functional outcome at 30 days post-operation.

**Table 1:** Baseline characteristics of ICH patients.

Characteristic	Total (*n* = 101)	Favorable Outcome (*n* = 42)	Poor Outcome (*n* = 59)	*p* value
Male, *n* (%)	69 (68.3%)	25 (59.2%)	44 (74.6%)	0.109
Age, IQR, Y	59.0 (53.5–66.0)	59.0 (54.8–67.0)	60.0 (53.0–66.0)	0.641
Admission systolic blood pressure, IQR, mmHg	177.0 (160.5–200.0)	175.5 (160.0–190.8)	177.0 (163.0–205.0)	0.200
Admission diastolic blood pressure, IQR, mmHg	102.0 (93.0–112.5)	103.0 (90.0–114.3)	102.0 (95.0–112.0)	0.354
Hypertension, *n* (%)	70 (69.3%)	29 (69.0%)	41 (69.5%)	0.962
Diabetes, *n* (%)	13 (12.9%)	3 (7.1%)	10 (16.9%)	0.147
Admission GCS score, IQR	9.0 (8.0–12.0)	10.0 (8.0–12.0)	8.0 (7.0–10.0)	0.031^*^
Interval from onset to admission, IQR, h	3.0 (2.0–5.0)	3.3 (2.0–6.3)	2.5 (1.5–4.0)	0.039^*^
Hematoma volume, IQR, mL	40.0 (28.3–60.6)	38.9 (18.8–55.1)	40.9 (30.5–62.0)	0.410
Hematoma location				0.042^*^
Basal ganglia, *n* (%)	50 (49.5%)	15 (35.7%)	35 (59.3%)	
Cerebral lobe, *n* (%)	35 (34.7%)	17 (40.5%)	18 (30.5%)	
Cerebellum, *n* (%)	16 (15.8%)	10 (23.8%)	6 (10.2%)	
Intraventricular hemorrhage, *n* (%)	55 (54.5%)	19 (45.2%)	36 (61.0%)	0.117
Subarachnoid hemorrhage, *n* (%)	24 (23.8%)	13 (31.0%)	11 (18.6%)	0.152
Surgical approach				0.338
Craniotomy, *n* (%)	77 (76.2%)	30 (71.4%)	47 (79.7%)	
MIPD, *n* (%)	24 (23.8%)	12 (28.6%)	12 (2%)	

GCS, Glasgow coma scale; MIPD, minimally invasive puncture and drainage; IQR, Interquartile range; *p < 0.05.

### 3.2. Univariate and multifactor analysis

In univariate analysis, admission GCS score, interval from onset to admission, hematoma location, NLR and PLR within 48 h after surgery were risk factors for 30-day postoperative prognosis in spontaneous ICH patients (*p* < 0.05). In addition, univariate analysis showed that age, sex, medical history, hematoma volume, combined ventricular hemorrhage, combined subarachnoid hemorrhage, surgical method, coagulation, serum potassium, serum glucose and other time points of NLR and PLR were not associated with prognosis (*p* > 0.05) (Tables 1, 2). In multivariate logistic analysis, NLR within 48 h after surgery was an independent risk factor for 30-day postoperative prognosis in spontaneous ICH patients (*p* < 0.05) (Table 3).

**Table 2:** Laboratory tests of ICH patients.

Hematological variables	Favorable outcome (*n* = 42)		Poor outcome (*n* = 59)		*p* value
	Mean ± SD	Med (IQR 25–75 %)	Mean ± SD	Med (IQR 25–75 %)	
Admission					
WBC (10^9^/L)	12.5 ± 3.3	11.8 (10.6–14.7)	12.4 ± 6.2	11.3 (8.6–14.6)	0.928
Neutrophils (10^9^/L)	10.6 ± 3.0	10.4 (8.2–12.8)	9.9 ± 4.8	9.3 (6.3–12.8)	0.370
Lymphocytes (10^9^/L)	1.3 ± 0.7	1.2 (0.8–1.6)	1.7 ± 1.4	1.2 (0.9–1.7)	0.165
Platelets (10^9^/L)	226.0 ± 66.6	220.5 (171.3–270.3)	216.0 ± 76.0	211.0 (163.0–246.0)	0.496
NLR1	9.9 ± 4.7	8.7 (6.5–12.6)	8.7 ± 5.9	7.8 (4.0–11.6)	0.276
PLR1	202.9 ± 79.2	206.3 (143.6–271.8)	174.3 ± 86.8	153.35 (105.7–222.2)	0.094
Glucose (mmol/L)	8.3 ± 3.1	7.2 (6.2–10.0)	9.3 ± 3.2	8.4 (6.7–11.4)	0.117
K^+^(mmol/L)	3.7 ± 0.6	3.8 (3.4–4.0)	3.7 ± 0.5	3.7 (3.4–4.1)	0.994
FIB(g/L)	3.2 ± 0.8	3.1 (2.7–3.7)	3.4 ± 1.4	3.1 (2.5–3.7)	0.410
PT(s)	12.8 ± 1.4	12.7 (12.0–13.2)	12.8 ± 0.9	12.7 (12.1–13.4)	0.767
APTT(s)	31.8 ± 5.5	30.1 (28.3–34.2)	31.6 ± 4.9	31.4 (27.5–34.5)	0.853
TT(s)	17.0 ± 1.6	17.3 (15.9–18.0)	17.0 ± 2.3	16.8 (15.9–18.0)	0.989
D-dimer(mg/l)	1.3 ± 2.7	0.6 (0.4–1.1)	1.2 ± 1.6	0.5 (0.3–1.1)	0.674
48 h after surgery					
WBC (10^9^/L)	13.1 ± 3.4	10.7 (10.4–15.1)	13.1 ± 3.2	13.2 (10.7–15.4)	0.963
Neutrophils (10^9^/L)	10.8 ± 2.9	10.8 (8.4–13.1)	11.2 ± 3.2	10.7 (8.6–14.0)	0.572
Lymphocytes (10^9^/L)	1.3 ± 0.6	1.1 (0.9–1.6)	1.1 ± 0.5	1.1 (0.7–1.5)	0.044^*^
Platelets (10^9^/L)	212.9 ± 58.2	207.0 (175.3–258.0)	206.2 ± 63.5	200.0 (163.0–246.0)	0.589
NLR2	9.3 ± 3.6	9.1 (6.6–11.8)	13.7 ± 10.4	10.5 (6.2–17.0)	0.004^*^
PLR2	183.9 ± 76.2	181.4 (111.7–245.4)	234.8 ± 139.3	184.0 (127.5–307.5)	0.021^*^
3-7d after surgery					
WBC (10^9^/L)	10.1 ± 2.7	9.9 (8.8–11.6)	11.2 ± 3.2	10.6 (9.1–13.7)	0.085
Neutrophils (10^9^/L)	7.7 ± 2.4	7.5 (5.8–9.4)	8.7 ± 2.9	7.9 (6.8–11.3)	0.072
Lymphocytes (10^9^/L)	1.4 ± 0.7	1.4 (1.1–2.0)	1.4 ± 0.7	1.3 (0.9–2.0)	0.828
Platelets (10^9^/L)	255.6 ± 82.2	267.5 (191.0–327.5)	232.4 ± 89.0	213.0 (162.0–279.5)	0.187
NLR3	6.5 ± 3.3	5.1 (3.5–8.1)	7.5 ± 4.8	6.6 (4.6–9.1)	0.236
PLR3	209.8 ± 107.4	165.8 (143.2–239.5)	195.2 ± 103.4	163.6 (127.0–234.8)	0.495

WBC, White blood cell; FIB, Fibrinogen; PT, Prothrombin time; APTT, Activated partial thromboplastin time; TT, Thrombin time; NLR1, admission neutrophil to lymphocyte ratio; PLR1, admission Platelet to lymphocyte ratio; NLR2, Neutrophil to lymphocyte ratio at 48 h after surgery; PLR2, Platelet to lymphocyte ratio at 48 h after surgery; NLR3, Neutrophil to lymphocyte ratio within 3–7 d after surgery; PLR3, Platelet to lymphocyte ratio within 3–7 d after surgery; Med, median; IQR, Interquartile range; SD, standard deviation; *p < 0.05.

**Table 3:** Univariate and multifactorial analysis of factors associated with poor postoperative prognosis in ICH patients.

	Univariate analysis		Multivariate analysis	
	OR (95 CI %)	*p* value	OR (95 CI %)	*p* value
Sex	0.051 (0.214–1.173)	0.111	0.475 (0.173–1.304)	0.149
Age	0.991 (0.956–1.028)	0.637	1.009 (0.966–1.055)	0.694
Admission GCS score	0.825 (0.690–0.985)	0.034	0.815 (0.663–1.055)	0.052
Interval from onset to admission	0.912 (0.834–0.998)	0.045	0.904 (0.817–1.001)	0.052
Basal ganglia hemorrhage	0.381 (0.168–0.863)	0.021	0.415 (0.164–1.054)	0.064
NLR2	1.093 (1.016–1.176)	0.017	1.147 (1.005–1.308)	0.042
PLR2	1.004 (1.000–1.008)	0.041	0.997 (0.990–1.005)	0.500

GCS, Glasgow coma scale; NLR2, Neutrophil to lymphocyte ratio at 48 h after surgery; PLR2, Platelet to lymphocyte ratio at 48 h after surgery; OR, Odds ratios; CI, Confidence interval.

### 3.3. Trend of NLR, PLR, and ROC curve

**Figure 1:**
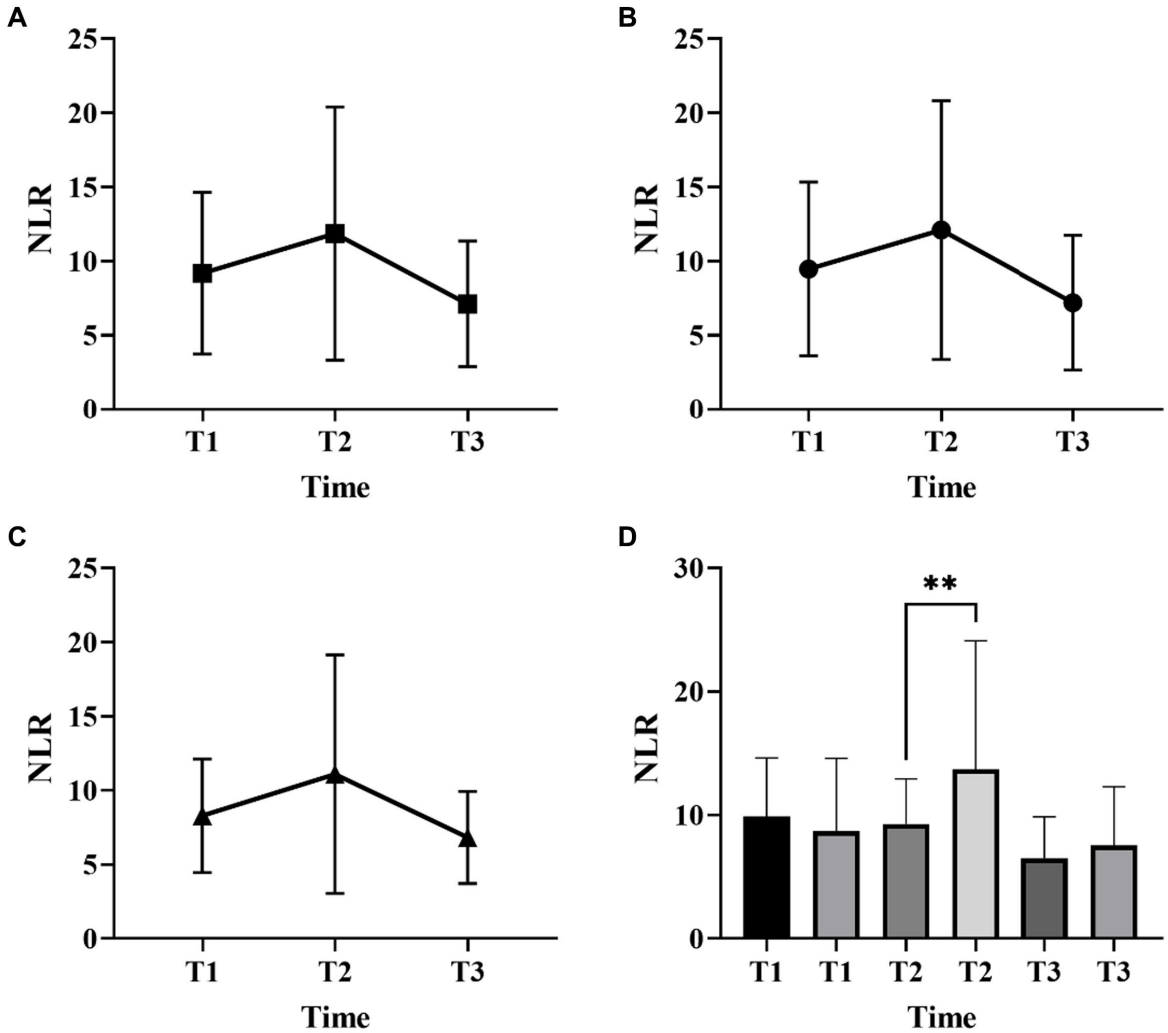
Neutrophil-to-lymphocyte ratio (NLR) changes over time in patients with ICH with two surgical methods (**A**, all patients; **B**, craniotomy; **C**, MIPD; **D**, comparison of NLR at different time points in ICH patients with favorable and poor prognosis; T1, at admission; T2, at 48 h after surgery; T3, within 3-7 days after surgery; **p < 0.05).

**Figure 2:**
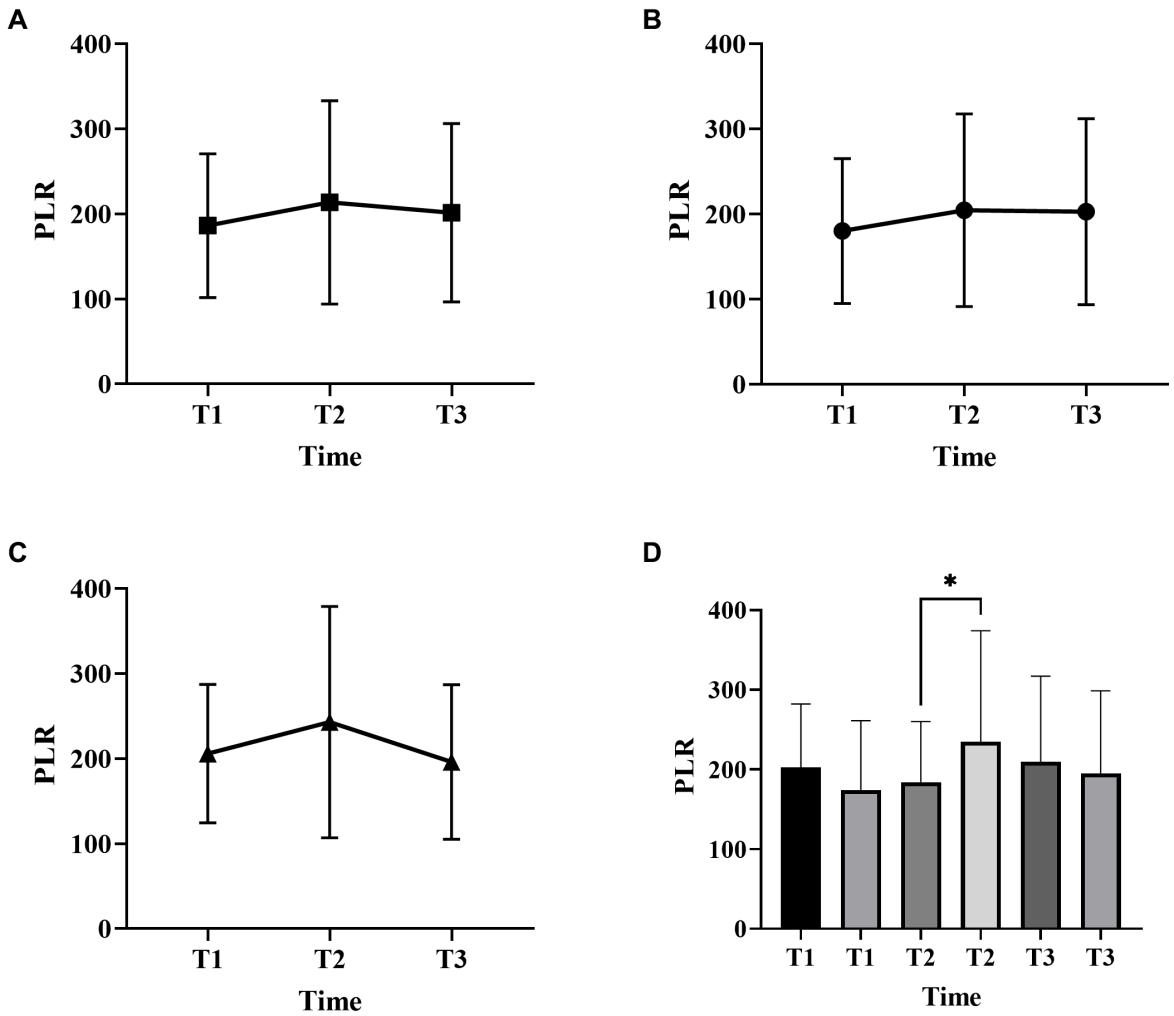
Platelet-to-lymphocyte ratio (PLR) changes over time in patients with ICH with two surgical methods (**A**, all patients; **B**, craniotomy; **C**, MIPD; **D**, comparison of PLR at different time points in ICH patients with favorable and poor prognosis; T1, at admission; T2, at 48 h after surgery; T3, within 3-7 days after surgery, *p < 0.05).

**Figure 3:**
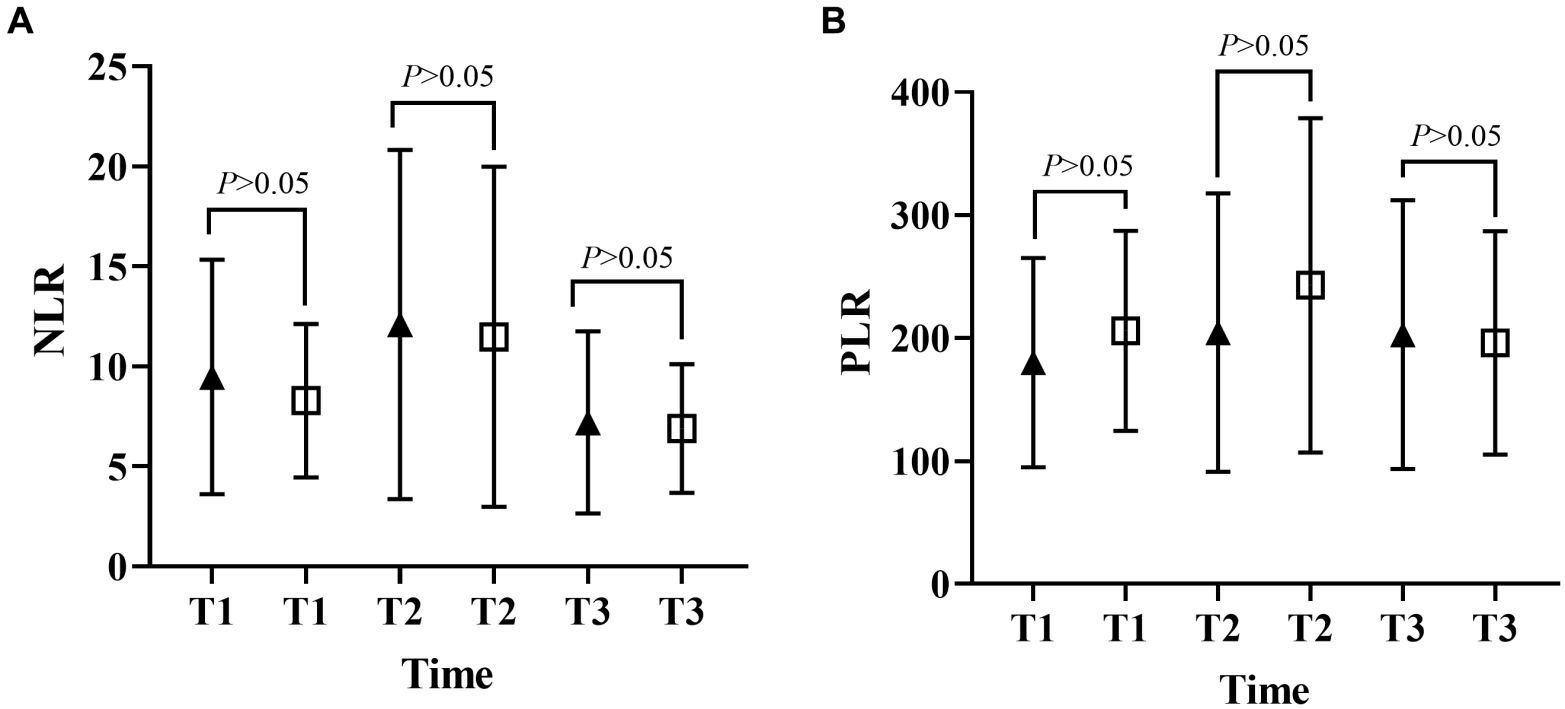
Differences in NLR and PLR between the open and MIPD groups in various time points (**A**, neutrophil-to-lymphocyte ratio; **B**, platelet-to-lymphocyte ratio; T1, at admission; T2, at 48 h after surgery; T3, within 3–7 days after surgery).

**Figure 4:**
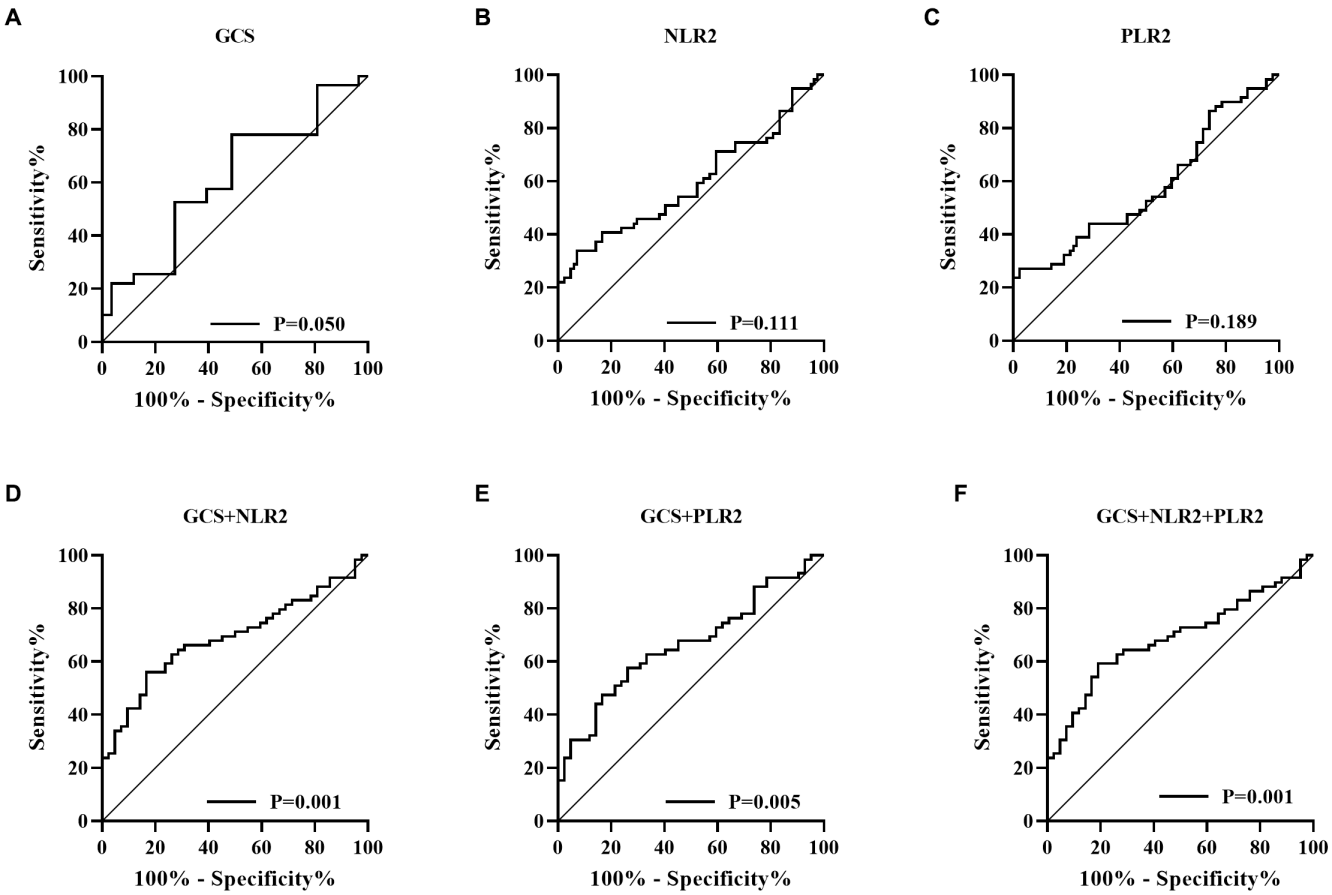
Receiver operating characteristic (ROC) curve (**A**, Glasgow coma scale; **B**, Neutrophil to lymphocyte ratio; **C**, Platelet to lymphocyte ratio; **D**, GCS combined with NLR2; **E**, GCS combined with PLR2; **F**, GCS combined with NLR2 and PLR2).

Figures 1, 2 described the changing patterns of NLR and PLR during the course of ICH disease, respectively. Figures 1A,2A showed that NLR and PLR exhibited a trend of increasing and then decreasing, and reached a peak within 48 h after surgery. The same trend was observed for the different surgical modalities (Figures 1B,C, 2B,C). The differences in NLR and PLR between the two surgical modalities (Figure 3) at admission, 48 h after surgery and 3–7 days after surgery were not significant (*p* > 0.05). The ROC curve results showed the area under the curve (AUC) was 0.593 for NLR2, 0.577 for PLR2, 0.690 for admission GCS score combined with NLR2, and 0.663 for admission GCS combined with PLR2 (Figure 4).

## 4. Discussion

In this study, NLR and PLR showed an increase followed by a decrease during spontaneous ICH and peaked within 48 h postoperatively. The two surgeries showed the same trend of change. This study found that measuring elevated levels of NLR and PLR within 48 h following surgery in spontaneous ICH patients is associated with a higher likelihood of poor prognosis. These results highlight the potential clinical importance of utilizing NLR and PLR as early biomarkers for prognosis in ICH patients following surgery. However, further research is needed to fully assess the reliability and efficacy of these biomarkers in predicting treatment outcomes.

The immune inflammatory response is closely associated with secondary brain injury after the occurrence of ICH. After hematoma formation, the blood–brain barrier (BBB) is disrupted, and the extravasated blood activates microglia, causing them to release large amounts of inflammatory factors and chemokines, which promote leukocyte infiltration in the surrounding brain tissue (18, 19). Neutrophils are the first immune cells to migrate through the damaged BBB to the hematoma (20). Neutrophils further damage the BBB by releasing myeloperoxidase, elastase and other inflammatory mediators, aggravating microvascular and brain tissue damage (20, 21). In a population-based study of ICH, neutrophil infiltration around brain tissue occurred as early as 8 h after hemorrhage, whereas in animals it occurred as early as 4 h after hemorrhage and peaked 2–3 days after onset (22, 23). In the acute phase of ICH, lymphopenia is considered to be the main marker of brain injury. The decrease in lymphocytes includes apoptosis and inactivation, mainly due to catecholamines and steroids produced by sympathetic and hypothalamic–pituitary–adrenal axis activation (24). Brain injury occurs after the infiltration of brain tissue around a large number of neutrophils and lymphocytes, the over-recruitment of immune cells make the body in a state of immunosuppression (25). After ICH occurs, platelets activate and accumulate around the vascular endothelial cells, prompting fibrinogen precipitation and collecting more platelets around the damaged brain tissue to make the blood in a hypercoagulable state, the aggregated platelets release numerous inflammatory factors to enhance the inflammatory response (26). The immune inflammatory response plays an important role in the process of spontaneous ICH, NLR and PLR are indicators of the overall and local inflammation and stress status of the organism. NLR and PLR have greater potential to become biological markers for estimating the severity of secondary brain injury and disease progression after ICH.

In this study, we found that NLR and PLR in spontaneous ICH patients showed a trend of increasing and then decreasing, peaking within 48 h after surgery. Previous studies had shown that peripheral neutrophils were significantly higher in ischemic and hemorrhagic stroke patients on admission, while lymphocytes usually remained unchanged or decreased. Thus, local and systemic inflammatory changes in patients with brain injury can be manifested through NLR. However, this similar trend is not reflected in stroke patients who receive non-surgical treatment. Most previous studies have focused on whether NLR can be a predictor of prognosis in certain diseases (6, 27–29), neglecting its trend in disease, especially in the surgical treatment of ICH. The reasons affecting this trend are unclear. We speculate that this may be related to the evolution of the hematoma. In the early stage, as the hematoma progresses, the inflammatory response gradually becomes stronger and the NLR increases, and as the disease improves, the inflammatory response gradually subsides and the NLR begins to decrease (30). In addition, the early surgical intervention relieves the mechanical compression of the brain tissue by the hematoma as well as prevents the massive release of inflammatory products (31, 32), which may also be influential factors in producing this change. There is a close relationship between the inflammatory response of the organism and the hypercoagulable state. The inflammatory response can contribute to a hypercoagulable state and thrombosis in the organism (33). The thrombosis can trigger inflammatory reactions in turn (34, 35). PLR is a platelet count to lymphocyte count ratio that reflects the interrelationship between inflammation and hypercoagulable state of the body (36), and plays an important role in the assessment of inflammatory response in many diseases (37, 38). Considering the effect of different surgical methods in inflammatory response, in this study we studied the trend of inflammatory indicators in craniotomy and MIPD separately. We found that NLR and PLR were more valuable in predicting the prognosis of spontaneous ICH patients within 48 h after surgery compared to other time points.

In this study, we performed a univariate analysis of NLR2 and PLR2, and we found that NLR2 and PLR2 were risk factors for prognosis in ICH patients (OR, 1.093, 95% CI 1.016 ~ 1.17.6; OR, 1.004, 95% CI 1.000 ~ 1.0008), whereas admission GCS score was a factor for patient prognosis (OR 0.825, 95% CI, 0.690 ~ 0.985).NLR and PLR play an important role in the prognosis of stroke and can be used as complementary indicators of prognosis (6, 7). NLR and PLR are simpler and easier to obtain than the high cost of CT exams. In addition, NLR and PLR can be dynamically observed during the course of the disease and the presence of peaks can help in early intervention and timely adjustment of the treatment plan, especially 48 h after surgery. In this study, we analyzed trends in NLR and PLR over the course of the disease from three groups: all patients, the craniotomy group, and the MIPD group, respectively. NLR and PLR in all three groups showed an increase followed by a decrease and reached a peak within 48 h after surgery. A retrospective study on surgical treatment of spontaneous ICH showed that patients treated with craniotomy had higher postoperative NLR than those treated with minimally invasive surgery, possibly because craniotomy damaged more brain tissue damage (31). However, in this study, we compared NLR levels between the craniotomy and MIPD groups at different time points, and we found no significant difference in NLR levels. We also compared the PLR levels of the two groups at different time points and found that the PLR in the MIPD group was slightly higher than that in the craniotomy group at 48 h after surgery, but there was no significant difference between the groups, which we believe may be related to the higher blood loss during the craniotomy. In addition, we found peak NLR ≥ 14.34 and 13.93,peak PLR ≥ 300.85 and 304.93 in the craniotomy and MIPD groups, suggesting a poorer prognosis. The threshold values in our study were similar to previous studies, which reported NLRs in the range of 12.97–14.46 (31, 39). There was considerable debate as to whether different surgical approaches could improve the early prognosis of ICH patients. In a prospective non-randomized comparative study investigating 198 spontaneous ICH patients, including 114 patients treated with craniotomy and 84 patients treated with MIPD, researchers found no difference in mortality between the two groups at 30 days and 1 year postoperatively, however neurological functional outcomes at 1 year were significantly higher in the MIPD group than in the craniotomy group (40). In addition, a large, high-quality meta-analysis showed that the timing of surgery was an important factor in patient survival outcomes, and that the differences in early mortality and improved long-term functional outcomes in ICH patients between different surgical approaches, including open and minimally invasive surgery, were not statistically significant (41). Our study also found no difference between craniotomy and MIPD in improving the early prognosis of ICH patients, but further follow-up and studies are needed for the long-term functional outcome of patients. The ROC curve results showed an AUC of 0.593 for NLR2 and 0.577 for PLR2, which were not particularly satisfactory. However, we combined the admission GCS scores separately and found an AUC of 0.690 and 0.663. In clinical work, it is important to obtain information on the inflammatory response of ICH patients during the disease process, which is easily achieved by detecting dynamic changes in NLR and PLR compared to serial CT examinations. The temporal trend of NLR and PLR allows neurosurgeons to better comprehend the disease course. Additionally, the emergence of peak NLR and PLR values is a reflection of the inflammatory and coagulation response of the body, which occurs independently of infection and therefore obviates the need for unnecessary antibiotic treatment. Moreover, NLR and PLR are essential prognostic indicators that aid neurosurgeons in evaluating ICH patient outcomes. Currently constructed prognostic models for ICH patients rely mainly on clinical information, such as admission GCS score and hematoma volume, ignoring the role of inflammatory indicators in predicting prognosis. We describe the pattern and value of NLR and PLR changes in spontaneous ICH patients after surgery, providing a new idea to explore the prognostic biological indicators of spontaneous ICH.

This study has some limitations. First, it was a small retrospective study with a limited sample size, and a large, multicenter retrospective study should be conducted in the future. Second, we failed to perform a specific stratified analysis of the bleeding site, which may have some limitations, and we will further investigate the specific site of bleeding in future work. Finally, due to the complexity of spontaneous ICH, future studies should focus more on leukocyte subunit immune function.

## 5. Conclusion

The NLR and PLR in spontaneous ICH patients showed a trend of increasing and then decreasing, reaching a peak within 48 h after surgery. Peak NLR and PLR were risk factors affecting the prognosis of spontaneous ICH patients 30 days after surgery, with peak NLR being an independent risk factor affecting prognosis.

## Data availability statement

The original contributions presented in the study are included in the article/supplementary material, further inquiries can be directed to the corresponding author.

## Ethics statement

The studies involving human participants were reviewed and approved by the Medical Ethics Review Committee of the Fukuang General Hospital of Liaoning Health Group. The patients/participants provided their written informed consent to participate in this study.

## Author contributions

JZ, CL, and YHo conceived and designed the study. JZ, CL, YHu, AY, YZ, and YHo collected and cleaned the data. JZ performed the data analysis and drafted the manuscript. YZ, YHo, YHu, and AY helped revise the manuscript. All authors read and approved the final manuscript.

## Funding

This study was funded by the Natural Science Foundation of Liaoning Province (No. 20180530024).

## Conflict of interest

The authors declare that the research was conducted in the absence of any commercial or financial relationships that could be construed as a potential conflict of interest.

## Publisher’s note

All claims expressed in this article are solely those of the authors and do not necessarily represent those of their affiliated organizations, or those of the publisher, the editors and the reviewers. Any product that may be evaluated in this article, or claim that may be made by its manufacturer, is not guaranteed or endorsed by the publisher.
